# Clinical outcomes and cost-effectiveness of brief guided parent-delivered cognitive behavioural therapy and solution-focused brief therapy for treatment of childhood anxiety disorders: a randomised controlled trial

**DOI:** 10.1016/S2215-0366(17)30149-9

**Published:** 2017-07

**Authors:** Cathy Creswell, Mara Violato, Hannah Fairbanks, Elizabeth White, Monika Parkinson, Gemma Abitabile, Alessandro Leidi, Peter J Cooper

**Affiliations:** a School of Psychology and Clinical Language Sciences, University of Reading, Reading, UK; b Statistical Services Centre, University of Reading, Reading, UK; c School of Mathematical, Physical, and Computational Sciences, University of Reading, Reading, UK; d Health Economics Research Centre, Nuffield Department of Population Health, University of Oxford, Oxford, UK; e Primary Child and Adolescent Mental Health Service, Oxford Health NHS Foundation Trust, Oxfordshire, UK; f Department of Psychology, Stellenbosch University, Stellenbosch, South Africa; g Department of Psychology, University of Cape Town, Cape Town, South Africa

## Abstract

**Background:**

Half of all lifetime anxiety disorders emerge before age 12 years; however, access to evidence-based psychological therapies for affected children is poor. We aimed to compare the clinical outcomes and cost-effectiveness of two brief psychological treatments for children with anxiety referred to routine child mental health settings. We hypothesised that brief guided parent-delivered cognitive behavioural therapy (CBT) would be associated with better clinical outcomes than solution-focused brief therapy and would be cost-effective.

**Methods:**

We did this randomised controlled trial at four National Health Service primary child and mental health services in Oxfordshire, UK. Children aged 5–12 years referred for anxiety difficulties were randomly allocated (1:1), via a secure online minimisation tool, to receive brief guided parent-delivered CBT or solution-focused brief therapy, with minimisation for age, sex, anxiety severity, and level of parental anxiety. The allocation sequence was not accessible to the researcher enrolling participants or to study assessors. Research staff who obtained outcome measurements were masked to group allocation and clinical staff who delivered the intervention did not measure outcomes. The primary outcome was recovery, on the basis of Clinical Global Impressions of Improvement (CGI-I). Parents recorded patient-level resource use. Quality-adjusted life-years (QALYs) for use in cost-utility analysis were derived from the Child Health Utility 9D. Assessments were done at baseline (before randomisation), after treatment (primary endpoint), and 6 months after treatment completion. We did analysis by intention to treat. This trial is registered with the ISCRTN registry, number ISRCTN07627865.

**Findings:**

Between March 23, 2012, and March 31, 2014, we randomly assigned 136 patients to receive brief guided parent-delivered CBT (n=68) or solution-focused brief therapy (n=68). At the primary endpoint assessment (June, 2012, to September, 2014), 40 (59%) children in the brief guided parent-delivered CBT group versus 47 (69%) children in the solution-focused brief therapy group had an improvement of much or very much in CGI-I score, with no significant differences between groups in either clinical (CGI-I: relative risk 1·01, 95% CI 0·86–1·19; p=0·95) or economic (QALY: mean difference 0·006, −0·009 to 0·02; p=0·42) outcome measures. However, brief guided parent-delivered CBT was associated with lower costs (mean difference −£448; 95% CI −934 to 37; p=0·070) and, taking into account sampling uncertainty, was likely to represent a cost-effective use of resources compared with solution-focused brief therapy. No treatment-related or trial-related adverse events were reported in either group.

**Interpretation:**

Our findings show no evidence of clinical superiority of brief guided parent-delivered CBT. However, guided parent-delivered CBT is likely to be a cost-effective alternative to solution-focused brief therapy and might be considered as a first-line treatment for children with anxiety problems.

**Funding:**

National Institute for Health Research.

## Introduction

Anxiety disorders are among the most common mental health disorders and, because of their high prevalence, persistence, and associated impairment, have a greater economic burden than any other mental health condition.^1^ Half of all lifetime cases emerge before age 12 years.^2^ Effective treatments for anxiety disorders in children exist;^3^ however, fewer than a third of children with an anxiety disorder access professional help.^4^ Both parental preferences^5^ and treatment side-effect profiles^6^ indicate the use of psychological treatments as the first-line treatment, yet evidence-based psychological treatments are typically lengthy (eg, 14–16 h-long sessions)^4^ and studies have mainly been done in specialist settings. Cost-effective psychological treatments suitable for routine clinical practice are needed.

Systematic evaluations of psychological interventions for childhood anxiety disorders have been limited to cognitive behavioural therapy (CBT).^7^ Although good evidence exists for the efficacy of CBT compared with waiting-list controls, few studies have compared this therapy with an active comparator and, when this has been done, the comparator has most commonly been an attention control condition rather than an established treatment.^3^ CBT can be effectively delivered in a brief form, whereby parents are supported in applying CBT principles. This approach is superior to a waiting-list comparison,^8^ with similar outcomes to CBT delivered in a more intensive traditional form.^9^ Indeed, brief guided parent-delivered CBT might be a cost-effective first-line treatment for childhood anxiety disorders. However, whether this therapy would be superior to a credible, alternative, brief psychological treatment remains unclear. We therefore aimed to compare the effectiveness and cost-effectiveness of two brief psychological treatments for childhood anxiety. We selected solution-focused brief therapy as the usual-care comparator because it is widely used in National Health Service (NHS) mental health settings, in which only a few sessions can be provided. Although this approach has not been evaluated with children with anxiety disorders specifically, our consultations revealed that it was the most commonly used approach for working with children with a range of difficulties, including anxiety, in the NHS services participating in this trial.

**Research in context**

**Evidence before this study**

Anxiety disorders are among the most common mental health disorders. Half of all lifetime cases emerge before age 12 years, affecting a substantial proportion of children worldwide. Good evidence exists to show that cognitive behavioural therapy (CBT) is an effective treatment for childhood anxiety, compared with waiting-list controls, with recovery rates around 60%. However, how CBT compares with other psychological therapies that are used in child mental health services remains unclear. Furthermore, most trials of CBT involve at least nine face-to-face treatment sessions, although this is not always practical in routine health settings in which resources are often scarce. We searched PsycINFO and MEDLINE from Jan 1, 2000, to April 1, 2016, with the search terms “anxi*”; “child”, “adolescent”, “paediatric”, “pediatric”, “youth”; “treatment”, “intervention”, “therapy”, “psychotherapy”; “bibliotherapy”, “computer*”, “technology”; and “randomi* controlled trial”, “clinical trial' to identify brief psychological interventions for childhood anxiety disorders. The most frequently evaluated brief treatment for childhood anxiety disorders was guided parent-delivered CBT (four studies) in which parents are supported by a therapist in working through a book that provides strategies to help them implement CBT strategies in their child's day-to-day life; however, none of the studies compared a brief psychological intervention with a credible control treatment, and none included an economic analysis.

**Added value of this study**

In this study, we compared the effect of two brief psychological interventions on both clinical and economic outcomes in children referred for problems with anxiety. 40 (59%) children in the brief guided parent-delivered CBT group versus 47 (69%) children in the solution-focused brief therapy group had an improvement of much or very much in Clinical Global Impression of Improvement score at the assessment after treatment, and 45 (66%) versus 49 (72%) children had an improvement at the 6 month follow-up assessment, with no significant differences between groups across both timepoints (relative risk 1·01, 95% CI 0·86–1·19; p=0·95). We did not find a significant difference in costs of providing the two therapies, both delivered with 5 h of therapist contact; however, when we considered the joint distribution of incremental mean costs and effects, brief guided parent-delivered CBT was likely to be cost-effective compared with solution-focused brief therapy.

**Implications of all the available evidence**

To our knowledge, this randomised controlled trial is the first to provide data for the outcomes of brief guided parent-delivered CBT compared with solution-focused brief therapy for childhood anxiety disorders in routine clinical practice. Although previous studies have shown that guided parent-delivered CBT is an effective treatment for childhood anxiety disorders compared with waiting-list controls, our findings show that it is no better than an alternative brief psychological treatment, solution-focused brief therapy, in terms of children's outcomes. Nonetheless, brief guided parent-delivered CBT might be a more cost-effective approach, building on previous studies that support its use as a low-intensity intervention to improve access to evidence-based treatments for childhood anxiety. Further studies are needed to examine how effective psychological treatments can be delivered at reduced costs without negatively affecting clinical outcomes and to establish the longer-term cost-benefits of intervention for children with these common, debilitating, and often chronic difficulties.

## Methods

### Study design and participants

We did this randomised controlled trial in children referred to four NHS primary child and adolescent mental health services in Oxfordshire, UK. Families were invited to participate if the child was aged 5–12 years with anxiety associated with clinical impairment as the primary presenting problem. We excluded children prescribed psychotropic medication, and parents or children with little understanding of English or with physical or intellectual impairment (including autism spectrum disorder) that would interfere with their ability to participate in assessments or treatment. Meeting diagnostic criteria for an anxiety disorder was not an inclusion criterion because we wanted to include all children referred for anxiety related problems.

The study was approved by the University of Reading (reference 12/02) and Oxford Health NHS Foundation Trust (reference 11/SC/0472) research ethics committees. Parents provided written consent and children written assent before randomisation.

### Randomisation and masking

Baseline assessments were done as part of routine clinical assessments before randomisation. The researcher who enrolled eligible participants then informed the study clinical supervisor (MP), who was independent of the recruitment process, who allocated children to trial groups, informed families, and allocated clinicians. Participants were randomly allocated (1:1), via a secure online minimisation tool developed by a researcher with no clinical involvement in the trial, to receive brief guided parent-delivered CBT or solution-focused brief therapy. The minimisation tool was developed to balance the two treatment groups for child age (in months), sex (male *vs* female), anxiety severity (Anxiety Disorders Interview Schedule Clinician Severity Rating; mild, moderate, severe), and level of parental anxiety (measured with the anxiety subscale of the 21 item Depression Anxiety and Stress Scale; mild, moderate, severe).^10^ The minimisation algorithm operated on the basis of 80% minimisation and 20% random allocation. However, the first nine patients were all allocated randomly to ensure that the algorithm was not predictable to research staff.

To ensure appropriate allocation concealment, the allocation sequence was retained on the secure online minimisation program, which was only accessible to the principal investigator (CC) and the clinical supervisor (MP) who allocated participants to clinicians. The allocation sequence was not accessible to the researcher enrolling participants or to study assessors. The trial adhered to procedures to maintain separation between research staff who measured outcomes and clinical staff who delivered the intervention. Research staff who obtained outcome measurements were masked to group allocation and clinical staff who delivered the intervention did not measure outcomes.

### Procedures

Families in both treatment groups received roughly 5 h of treatment, which was audio recorded to allow for checks of treatment adherence.

For brief guided parent-delivered CBT, as in previous studies,^8^, ^11^ parents were given a self-help book^12^ and received up to eight weekly sessions of therapist supported brief guided parent-delivered CBT (5 h total contact). Four of these sessions were done face to face (about 45 min) and four were brief telephone reviews (about 15 min). The treatment focused on psychoeducation about childhood anxiety, identification and testing of anxious thoughts, graded exposure, and problem solving. Parents completed homework tasks between sessions, both independently and with their child. The therapists followed a treatment manual specific to this programme,^13^ which instructed them in how to support and encourage parents to work through the self-help book, rehearse skills, and solve any problems that arose.

Solution-focused brief therapy is a form of counselling that is future-focused and works with the strengths and resources of the individual to build solutions. The delivery format was based on usual practice within the participating services and consultations with an expert adviser from BRIEF (a leading international centre for solution-focused brief therapy training). Solution-focused brief therapy comprised an initial face-to-face session with the parent and child to initiate treatment (60 min), four face-to-face sessions of solution-focused brief therapy with the child (four 45 min sessions), and a final session with the child and parent (60 min; 5 h total contact). Therapists followed a manualised approach that was adapted from a solution-focused practice manual, and consistent with the European Brief Therapy Association practice definition.^14^

Therapists were primary mental health workers employed within participating services with a range of backgrounds, including health visiting, nursing, occupational therapy, social and youth work, and clinical psychology and psychology graduates; and with varying degrees of experience in working with parents and children (none to several years). Therapists received 2 days of training in each treatment approach and fortnightly supervision throughout the trial. They were each allocated to deliver one treatment for the first half of the trial and the other treatment for the second half (with training before each treatment phase). Participants were allocated to receive treatment with the next available clinician who was assigned to the appropriate treatment within their locality team.

### Outcomes

Assessment points were at baseline (before randomisation), after treatment (primary endpoint), and 6 months after treatment completion. Assessments were done in participants' homes (unless this was not possible or wanted, and an alternative venue [eg, school] was organised).

The primary outcome was clinician-rated recovery. The primary indicator of recovery was an improvement of much or very much in the child's difficulties, on the basis of Clinical Global Impression of Improvement (CGI-I),^15^ as determined by independent assessors. The CGI-I was established on the basis of the parent report and child report on the Anxiety Disorders Interview Schedule, child and parent version (ADIS-c/p),^16^ which assesses the frequency and severity of symptoms of DSM-IV anxiety disorders and associated interference. The ADIS-c/p has not been validated in a child-report form in children younger than 7 years; therefore, parents of children aged 5–6 years completed the full ADIS and children were administered a brief version.

Secondary outcome measures were clinical severity ratings from the ADIS-c/p, and parent-report and child-report questionnaires of anxiety symptoms and interference. Symptoms of anxiety were assessed among children aged 7 years and older with the Spence Children's Anxiety Scale-child and parent version (SCAS-c/p)^17^— a parent-report and child-report questionnaire validated with children of this age.^18^, ^19^ All children completed the Koala Fear Questionnaire, which has been validated in children aged 4 years and older.^20^ Interference associated with anxiety within school, social, and home and family domains was assessed with the Child Anxiety Impact Scales, child and parent version (CAIS-c/p).^21^, ^22^ The CAIS-c was used in children from age 7 years; we removed two items about dating that are not typically applicable at this age. For children who met diagnostic criteria for a current anxiety disorder, we additionally examined the dichotomous outcomes of recovery from primary anxiety disorder and recovery from all anxiety disorders, on the basis of the ADIS-c/p.

We adopted a societal perspective in assessment of resource use and costs. Treatment-related health care and other patient-level resource-use data were collected over three separate time periods (3 months before baseline assessment, baseline to after treatment, after treatment to 6 month follow-up) on a modified Client Service Receipt Inventory form^23^ by use of parent-report patient-health diaries. These data included all health, social care, non-NHS (eg, educational) cost-generating services, and lost leisure and productivity time estimates for parents.

We assessed child quality of life with the Child Health Utility 9D (CHU-9D)^24^—a paediatric generic preference-based measure of health-related quality of life, completed by children and their main caregiver. Preference weights for the CHU-9D valuation were obtained from a UK general population sample.^24^ We used the Euroqol-5D-Youth version^25^ in sensitivity analyses. Both measures allow for calculation of quality-adjusted life-years (QALYs) for use in cost-utility analysis.

### Statistical analysis

Data collection was in pen and paper form and data were initially entered in to SPSS datasheets (version 22).

Probable outcomes for CGI-I were not available for solution-focused brief therapy for childhood anxiety, so the study was powered on the basis of two considerations: (1) a two-thirds difference in the proportion of recovered children, because this outcome could be considered justification for service changes required to adopt a new approach; and (2) a meta-analysis of mixed outcomes of solution-focused brief therapy^26^ reported an effect size of 0·26 for internalising problems (including anxiety) compared with 0·52 in feasibility work using guided parent-delivered CBT.^11^ A sample size of 136 would provide 80% power to detect either difference at a two-sided 5% significance level.

A full data analysis plan was produced by the statistician (HF) and principal investigator (CC) before database lock. Analyses were done on a complete case basis on unmasked data. We did analyses by intention to treat. For primary and secondary analyses, mixed models were fitted to each outcome including the fixed effects of treatment; timepoint (categorical: before, after, 6 months); treatment by timepoint interaction; child's sex, age, and primary disorder type; and parental anxiety level (at the minimisation stage). For the primary analysis (CGI-I), a log-binomial mixed model was fitted including the additional fixed effect baseline severity of the child's primary anxiety disorder (ADIS clinical severity ratings). Linear mixed models were fitted for the continuous secondary analyses, which also included the fixed effect baseline total score for the endpoint being analysed. The baseline severity of the child's primary anxiety disorder was not included in the analysis because of its high correlation with the baseline total score of the endpoints being analysed. Additional analyses were done to model the binary outcomes: free from primary diagnosis, and free from all anxiety diagnoses. The same underlying model as the primary CGI-I analysis was fitted to these endpoints. Repeated measurements were taken into account by allowing measurements taken on the same child to be correlated in all models. Relative risks or differences were estimated to compare categorical fixed effects. Parameter estimates were obtained for continuous fixed effects. We calculated 95% CIs for all estimates.

Lack of convergence in the generalised mixed models for CGI-I, free from primary, and free from all diagnoses resulted in the fixed effects of child's type of primary disorder, baseline severity of the child's primary anxiety disorder, and parental anxiety level, being removed from these models. The fixed effect of sex was also removed from the model of free from primary diagnosis. Analyses of clinical outcomes were done with SAS (version 9.3).

For the base-case economic evaluation, we adopted a cost-utility analysis framework to assess the cost-effectiveness of brief guided parent-delivered CBT compared with solution-focused brief therapy from a societal perspective. We followed current best-practice methods for conducting and reporting economic evaluation alongside trials.^27^ Costs were expressed in pounds sterling (£) in 2013–14 prices. In view of the short timeframe of the trial and follow-up, discounting was not applied to costs or effects. An intention-to-treat approach was adopted in the base-case analysis. Missing data for resource use and health outcomes were imputed by use of mean imputation for missing values deemed highly deterministic (eg, face-to-face therapists contact), and multiple imputation for other resources (eg, use of medications), under the assumption of missing at random.^28^ For each trial participant, all components of treatment costs, stratified by category of resource use, and other wider societal costs (educational services, travel costs, time off school or, for the main caregiver, work) were computed by multiplying units of resource use by their unit costs (appendix). These values were then summed to obtain a total cost for each patient.

A deviation from the original protocol was that days off school were considered a consequence rather than an outcome and were included in the costs part of the economic analysis, in line with relevant economic literature. This decision was made in April, 2015, and was orally agreed with the principal investigator (CC). Independent members of the trial steering committee did not consult on this deviation as it was not considered to be substantial by the trial management group (and there was no independent health economic expertise on the trial steering committee).^29^

Effects were identified and measured using QALYs, derived from the CHU-9D child report in the base-case analysis. Incremental mean costs and effects, and the associated 95% CIs, were estimated comparing the two intervention groups. Incremental cost-effectiveness ratios were estimated and reported where relevant. Uncertainty in the cost-effectiveness results was analysed by use of cost-effectiveness acceptability curves over a range of potential threshold values that the health system might be willing to pay for an additional QALY gained.^30^ Furthermore, we did ten sensitivity analyses to examine robustness of the base-case analysis results. We additionally did a cost-effectiveness analysis using the main clinical outcome of CGI-I.

Analyses of economic outcomes were done with STATA (version 13.1). This trial was registered with the ISCRTN registry, number ISRCTN07627865, on March 13, 2012.

### Role of the funding source

The funder of the study reviewed the study proposal, awarded funding, and monitored the conduct of the study. The funders had no role in study design, data collection, data analysis, data interpretation, or writing of the report. The corresponding author had full access to all the data in the study and had final responsibility for the decision to submit for publication.

## Results

**Figure 1:**
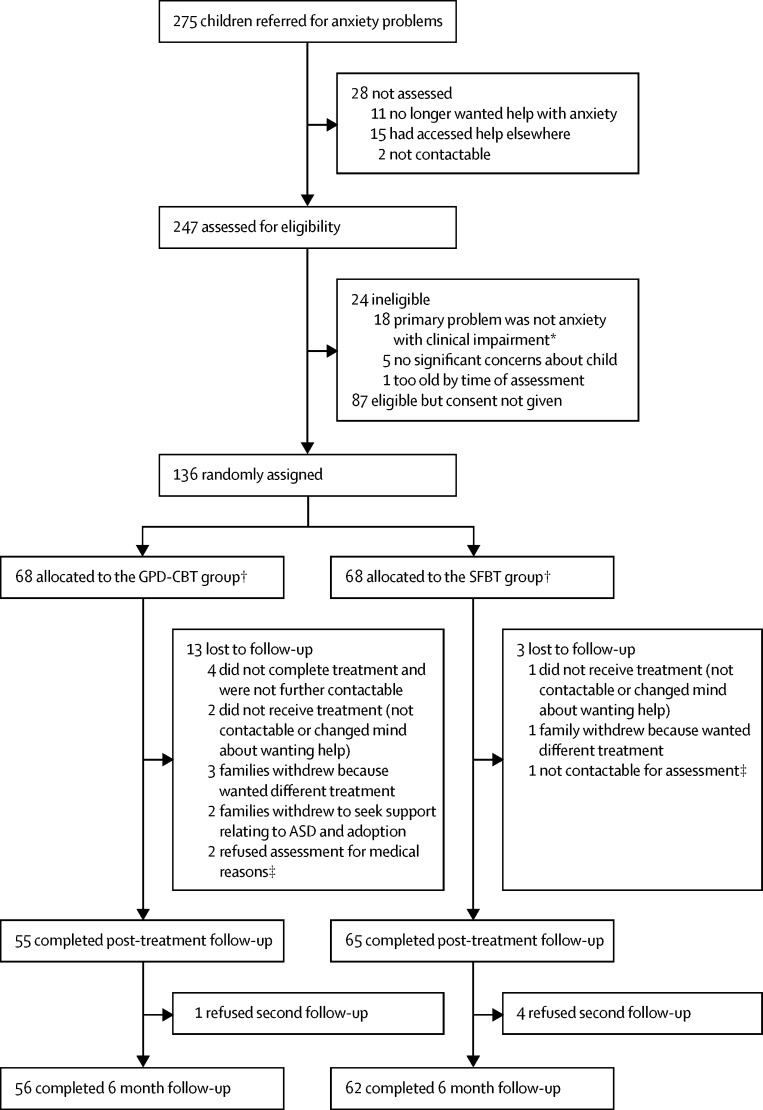
Trial profile GPD-CBT=guided parent-delivered cognitive behavioural therapy. SFBT=solution-focused brief therapy. ASD=autism spectrum disorder. *Oppositional defiant disorder (n=5), dysthymia (n=1), obsessive compulsive disorder (n=3), major depressive disorder (n=2), post-traumatic stress disorder (n=3), autistic spectrum disorder (n=3), and tic disorder (n=1). †One child assigned to solution-focused brief therapy received brief guided parent-delivered CBT in error; as such, the analysis was repeated on the treatment-received population (n=69 in the GPD-CBT group and n=67 in the SFBT group). ‡Completed 6 month follow-up assessment.

Between March 23, 2012, and March 31, 2014, we randomly assigned 136 children to receive brief guided parent-delivered CBT (n=68) or solution-focused brief therapy (n=68; figure 1). One child assigned to the solution-focused brief therapy group received brief guided parent-delivered CBT in error; as such, the analysis was repeated on the treatment-received population (n=69 in the brief guided parent-delivered CBT group and n=67 in the solution-focused brief therapy group), with no changes in the overall conclusions. The timing of the assessments before randomisation, after treatment (June, 2012, to September, 2014), and 6 months after treatment completion (November, 2012, to December, 2014) did not differ significantly between groups (appendix). Baseline characteristics were similar between groups (table 1). 122 (90%) participants met criteria for a diagnosis of current anxiety disorder, despite this not being an inclusion criterion.

**Table 1:** Baseline demographic and clinical characteristics

		**GPD-CBT (n=68)**	**SFBT (n=68)**
Age (months)		111·31 (21·93)	109·74 (26·76)
Sex			
	Female	36 (53%)	36 (53%)
	Male	32 (47%)	32 (47%)
Ethnic group			
	White British	62 (91%)	65 (96%)
	Other*	6 (9%)	3 (4%)
Two-parent household		60 (88%)	56 (82%)
Employment status of participating parent			
	Unemployed	18 (26%)	15 (22%)
	Part-time work	38 (56%)	36 (53%)
	Full-time work	11 (16%)	15 (22%)
	Employed–unspecified	0	2 (3%)
	Missing	1 (1%)	0
Household income (£)			
	<10 000	5 (7%)	6 (9%)
	10–15 000	5 (7%)	3 (4%)
	15–20 000	8 (12%)	6 (9%)
	20–30 000	8 (12%)	12 (18%)
	30–40 000	8 (12%)	12 (18%)
	40–50 000	12 (17%)	9 (13%)
	>50 000	20 (30%)	18 (26%)
	Missing	2 (3%)	2 (3%)
Education level of participating parent			
	Higher education	27 (40%)	22 (32%)
Socioeconomic status (highest of parent or partner)			
	Associate professional or technical	42 (62%)	40 (59%)
Anxiety level of participating parent (DASS-A)			
	Normal (0–3)	51 (75%)	50 (74%)
	Borderline (4–7)	13 (19%)	13 (19%)
	High (≥8)	4 (6%)	5 (7%)
Primary diagnosis of child anxiety disorder			
	Separation anxiety disorder	10 (15%)	13 (19%)
	Social anxiety disorder	6 (9%)	8 (12%)
	Generalised anxiety disorder	33 (49%)	36 (53%)
	Specific phobia	17 (25%)	10 (15%)
	Other†	2 (3%)	1 (1%)
ADIS-c/p clinical severity rating			
	Mild (3)	6 (9%)	8 (12%)
	Moderate (4–5)	31 (46%)	32 (47%)
	Severe (6–8)	31 (46%)	41 (28%)

Data are mean (SD) or n (%), unless otherwise stated. GPD-CBT=guided parent-delivered-cognitive behavioural therapy. SFBT=solution-focused brief therapy. DASS-A=Depression Anxiety and Stress Scales-Anxiety subscale. ADIS-c/p=Anxiety Disorders Interview Schedule for DSM-IV-child and parent version.

* GPD-CBT: white Irish (n=1), any other white background (n=1), white and black Caribbean (n=3), and Pakistani (n=1); SFBT: white and black African (n=1), any other mixed background (n=1), Caribbean (n=1).

† Panic disorder with or without agoraphobia or anxiety disorder not otherwise specified.

Before delivering treatment, 17 (90%) of 19 therapists reported that they used solution-focused brief therapy and 19 (100%) used CBT at least sometimes. Therapists varied in whom they typically worked with in their routine practice with children with anxiety problems (work with children, parents, or both: sometimes, three [17%], five [28%], two [11%]; frequently, eight [44%], nine [50%], eleven [61%]; or always, seven [39%], four [22%], five [28%], respectively [data missing for one clinician]). Recordings of a random sample of 52 treatment sessions were rated for treatment adherence by raters masked to treatment group. Session content was clearly differentiated, with brief guided parent-delivered CBT having more brief guided parent-delivered CBT-allowable content than solution-focused brief therapy (*t*[50]=16·88; p<0·0001) and solution-focused brief therapy having more solution-focused brief therapy-allowable content than brief guided parent-delivered CBT (*t*[50]=22·31; p<0·0001).

Inter-rater reliability was high for the primary outcome of CGI-I (mean κ=0·92, 95% CI 0·86–1·0). 40 (59%) children in the brief guided parent-delivered CBT group versus 47 (69%) children in the solution-focused brief therapy group had an improvement of much or very much in CGI-I score at the assessment after treatment, and 45 (66%) versus 49 (72%) children had an improvement at the 6 month follow-up assessment (table 2), with no significant differences between groups across both timepoints (relative risk 1·01, 95% CI 0·86–1·19; p=0·95; table 3).

**Table 2:** Primary, secondary, and economic outcome measures

		**GPD-CBT (n=68)**	**SFBT (n=68)**
**Dichotomous measures of outcome**			
CGI-I much or very much improved			
	After treatment	40 (59%)	47 (69%)
	6 month follow-up	45 (66%)	49 (72%)
Free of primary anxiety disorder diagnosis			
	After treatment	33 (50%)	40 (59%)
	6 month follow-up	47 (69%)	46 (68%)
Free of all anxiety disorder diagnoses			
	After treatment	25 (37%)	30 (44%)
	6 month follow-up	34 (50%)	35 (51%)
**Continuous measures of outcome**			
ADIS-c/p clinical severity rating of primary diagnosis*			
	Baseline	5·10 (1·08); n=68	5·13 (1·02); n=68
	After treatment	2·60 (2·00); n=55	2·77 (1·94); n=65
	6 month follow-up	1·88 (1·90); n=56	1·89 (1·96); n=62
SCAS-c*			
	Baseline	38·61 (20·31); n=54	34·15 (20·06); n=60
	After treatment	31·49 (24·09); n=45	24·36 (18·28); n=59
	6 month follow-up	23·77 (18·96); n=44	19·55 (17·08); n=55
SCAS-p*			
	Baseline	35·41 (17·55); n=66	32·56 (13·54); n=66
	After treatment	24·76 (12·88); n=51	24·44 (13·61); n=62
	6 month follow-up	22·41 (11·58); n=51	23.05 (14.67); n=58
CAIS-c (25 items)*			
	Baseline	15·95 (11·89); n=55	16·85 (11·44); n=62
	After treatment	10·55 (12·02); n=44	10·39 (9·80); n=59
	6 month follow-up	9·68 (13·56); n=41	7·88 (7·90); n=52
CAIS-p (25 items)*			
	Baseline	20·43 (12·24); n=64	19·90 (11·47); n=62
	After treatment	13·59 (12·41); n=51	12·04 (9·91); n=57
	6 month follow-up	10·84 (11·79); n=49	12·27 (10·93); n=55
KFQ-c*			
	Baseline	59·93 (11·33); n=68	58·68 (12·04); n=68
	After treatment	52·96 (14·22); n=50	51·56 (13·17); n=62
	6 month follow-up	50·23 (11·75); n=47	47·40 (12·94); n=58
CHU-9D-c†			
	Baseline	0·87 (0·09); n=64	0·88 (0·09); n=65
	After treatment	0·90 (0·10); n=49	0·90 (0·09); n=59
	6 month follow-up	0·91 (0·08); n=47	0·91 (0·08); n=55
CHU-9D-p†			
	Baseline	0·85 (0·10); n=46	0·89 (0·07); n=54
	After treatment	0·92 (0·07); n=53	0·92 (0·07); n=53
	6 month follow-up	0·93 (0·07); n=48	0·92 (0·07); n=56
EQ-5D-Y-c†			
	Baseline	0·82 (0·15); n=63	0·80 (0·20); n=67
	After treatment	0·88 (0·21); n=48	0·86 (0·21); n=61
	6 month follow-up	0·87 (0·19); n=47	0·91 (0·16); n=57

Data are n (%) or mean (SD); n. GPD-CBT=guided parent-delivered-cognitive behavioural therapy. SFBT=solution-focused brief therapy. CGI-I=Clinician Global Impression-Improvement. ADIS-c/p=Anxiety Disorders Interview Schedule for DSM-IV child and parent version. SCAS-c=Spence Children's Anxiety Scale-child report. SCAS-p=Spence Child Anxiety Scale-parent report. CAIS-c=Child Anxiety Impact Scale-child report. CAIS-p=Child Anxiety Impact Scale-parent report. KFQ-c=Koala Fear Questionnaire-child report. CHU-9D-c=Child Health Utility-9D-child report. CHU-9D-p=Child Health Utility-9D-parent report. EQ-5D-Y=EuroQol-5D-Youth version-child report.

* A higher score indicates higher symptoms or severity.

† A higher score indicates higher quality of life.

**Table 3:** Primary analysis results

		**CGI-I**		**Free from primary diagnosis**		**Free from all anxiety diagnoses**	
		Relative risk (95% CI)	p value*	Relative risk (95% CI)	p value*	Relative risk (95% CI)	p value*
Treatment							
	GPD-CBT *vs* SFBT (ref)	1·013 (0·863–1·189)	0·95	1·091 (0·915–1·300)	0·64	1·059 (0·838–1·340)	0·78
Timepoint							
	After treatment (ref) *vs* 6 month follow-up	0·883 (0·788–0·990)	0·036	0·773 (0·668–0·894)	0·0014	0·723 (0·601–0·870)	0·0020
Treatment by timepoint interaction		0·964 (0·733–2·125)	0·76	0·873 (0·402–1·437)	0·37	0·923 (0·650–1·955)	0·67
	GPD-CBT *vs* SFBT (ref) after treatment	0·977 (0·782–1·221)	0·83	0·979 (0·733–1·307)	0·88	0·977 (0·690–1·441)	0·98
	GPD-CBT *vs* SFBT (ref) at 6 month follow-up	1·013 (0·863–1·189)	0·87	1·121 (0·930–1·352)	0·23	1·079 (0·842–1·385)	0·54
Sex†							
	Male *vs* female	0·794 (0·662–0·951)	0·0093	..	..	0·909 (0·717–1·152)	0·50
Age		1·004 (0·968–1·042)	0·91	1·018 (0·965–1·074)	0·404	0·974 (0·916–1·037)	0·49

CGI-I=Clinician Global Impression-Improvement. GPD-CBT=guided parent-delivered cognitive behavioural therapy. SFBT=solution-focused brief therapy.

* p values from the fixed effects of the generalised (log-binomial) mixed models.

† The fixed effect sex was removed from the model of free from primary diagnosis.

Inter-rater reliability was good for both anxiety disorder diagnoses (κ=0·86, 95% CI 0·75–0·98) and clinical severity ratings (intraclass correlation coefficient 0·91, 95% CI 0·79–1·0) from the ADIS-c/p. No secondary outcome differed significantly between groups; however, all secondary endpoints except for CAIS-c reduced significantly over time (Table 2, Table 4). No treatment-related or trial-related adverse events were reported in either group.

**Table 4:** Secondary analysis results

		**KFQ-c**	**SCAS-c**	**SCAS-p**	**CAIS-c**	**CAIS-p**	**ADIS-c/p CSR**
Treatment							
	GPD-CBT *vs* SFBT (ref)	0·817 (−2·785 to 4·418); p=0·65	4·944 (−0·932 to 10·820); p=0·098	–2·072 (−5·733 to 1·590); p=0·27	1·129 (−2·668 to 4·927); p=0·56	–0·832 (−4·008 to 2·344); p=0·60	–0·177 (−0·783 to 0·429); p=0·56
Timepoint							
	After treatment (ref) *vs* 6 month follow–up	2·737 0·847 to 4·627); p=0·0049	5·267 (2·491 to 8·042); p=0·0003	2·939 (1·078 to 4·798); p=0·0022	0·617 (−1·106 to 2·340); p=0·48	1·931 (0·411 to 3·451); p=0·013	0·765 (0·432 to 1·097); p<0·0001
Treatment by timepoint interaction		–1·917 (−5·613 to 1·778); p=0·31	3·72 (−1·596 to 9·053); p=0·17	1·99 (−1·617 to 5·602); p=0·28	–1·665 (−4·963 to 1·632); p=0·32	1·839 (−1·111 to 4·789); p=0·22	–0·210 (−0·860 to 0·440); p=0·52
	GPD-CBT *vs* SFBT (ref) after treatment	–0·142 (−3·99 to 3·703); p=0·94	6·808 (0·312 to 13·305); p=0·040	–1·705 (−4·885 to 2·734); p=0·57	0·296 (−3·414 to 4·008); p=0·87	0·087 (−3·521 to 3·696); p=0·96	–0·282 (−0·958 to 0·394); p=0·41
	GPD-CBT *vs* SFBT (ref) at 6 month follow-up	1·775 (−2·46 to 6·015); p=0·41	3·08 (−3·33 to 9·487); p=0·34	–3·067 (−7·406 to 1·271); p=0·16	1·962 (−2·568 to 6·493); p=0·39	–1·751 (−5·143 to 1·640); p=0·30	–0·072 (−0·770 to 0·627); p=0·84
Child's baseline endpoint score		0·764 (0·597 to 0·931); p<0·0001	0·637 (0·487 to 0·787); p<0·0001	0·602 (0·478 to 0·726); p<0·0001	0·456 (0·305 to 0·608); p=<0·0001	0·724 (0·570 to 0·878); p<0·0001	0·515 (0·221 to 0·809); p=0·0007
Sex							
	Male (ref) *vs* female	–1·440 (−5·152 to 2·272); p=0·44	–4·340 (−10·372 to 1·693); p=0·16	2·022 (−1·707 to 5·750); p=0·29	–2·587 (−6·257 to 1·083); p=0·17	–0·296 (−3·527 to 2·934); p=0·86	0·118 (−0·499 to 0·734); p=0·71
Age		0·018 (−1·017 to 1·054); p=0·97	0·364 (−1·628 to 2·355); p=0·72	–0·040 (−1·025 to 0·944); p=0·94	–0·132 (−1·304 to 1·039); p=0·82	0·632 (−0·271 to 1·534); p=0·17	–0·141 (−0·309 to 0·028); p=0·1005
Parental level of anxiety		p=0·68*	p=0·53*	p=0·29*	p=0·80*	p=0·12*	p=0·99*
	Borderline (ref) *vs* normal	–0·957 (−5·733 to 3·819); p=0·69	–2·340 (−10·146 to 5·467); p=0·55	–3·523 (−8·334 to 1·288); p=0·15	–0·115 (−5·046 to 4·818); p=0·96	–4·135 (−8·432 to 0·163); p=0·059	0·000† (−0·820 to 0·820)
	Borderline (ref) *vs* high	–3·600 (−11·715 to 4·516); p=0·38	–7·075 (−19·447 to 5·297); p=0·26	–4·980 (−13·208 to 3·248); p=0·23	–2·374 (−10·162 to 5·414); p=0·55	–0·075 (−8·383 to 8·233); p=0·99	0·078 (−1·319 to 1·475); p=0·91
Child's primary anxiety disorder		p=0·54*	p=0·92*	p=0·023*	p=0·55*	p=0·077*	p=0·063*
	GAD (ref) *vs* SAD	–2·844 (−7·893 to 2·205); p=0·27	–2·701 (−11·151 to 5·749); p=0·53	2·549 (−2·585 to 7·682); p=0·33	2·678 (−2·538 to 7·894); p=0·31	3·559 (−1·109 to 8·226); p=0·13	0·488 (−0·366 to 1·342); p=0·26
	GAD (ref) *vs* social phobia	2·384 (−4·498 to 9·267); p=0·49	0·207 (−13·541 to 13·955); p=0·98	–5·200 (−12·182 to 1·781); p=0·14	–3·086 (−10·375 to 4·203); p=0·40	–4·188 (−10·208 to 1·831); p=0·17	–0·794 (−1·889 to 0·302); p=0·15
	GAD (ref) *vs* other	0·698 (−3·782 to 5·178); p=0·76	0·410 (−6·652 to 7·472); p=0·91	−5·770 (−10·422 to −1·118); p=0·016	1·055 (−3·382 to 5·492); 0·64	–2·299 (−6·311 to 1·712); p=0·26	–0·676 (−1·454 to 0·102); p=0·088

Data are estimate (95% CI); p value. KFQ-c=Koala Fear Questionnaire-child report. SCAS-c=Spence Children's Anxiety Scale-child report. SCAS-p=Spence Child Anxiety Scale–parent report. CAIS-c=Child Anxiety Impact Scale–child report. CAIS-p=Child Anxiety Impact Scale-parent report. ADIS-c/p=Anxiety Disorders Interview Schedule for DSM-IV child and parent version. CSR=Clinical Severity Rating. GPD-CBT=guided parent-delivered cognitive behavioural therapy. SFBT=solution-focused brief therapy. GAD=generalised anxiety disorder. SAD=social anxiety disorder.

* p values assessing the overall effect of categorical factors with three or more levels do not have a corresponding single effect measure so estimates and 95% CIs are not presented.

† Final figure as provided by the statistical programme.

QALYs gained over the trial period did not differ significantly between groups in the base-case analysis (mean difference 0·006, 95% CI −0·009 to 0·02; p=0·42). The mean societal cost for children was £1494 (SD 1107·79) for patients in the brief guided parent-delivered CBT and £1942 (1590·91) for patients in the solution-focused brief therapy group (mean difference −£448, 95% CI −934 to 37; p=0·070). The main drivers of the lower cost of the CBT intervention were treatment costs (mean difference −£133, 95% CI −204 to −63; p<0·0001), and time off school, work, or leisure time for children and parents (−£200, −386 to −13; p=0·036). In particular, despite the fact that both treatments lasted a similar time, cost savings occurred in the therapists' travel costs associated with the treatment delivery, both in terms of opportunity cost of their time (ie, time that they could have spent in other activities) and mileage cost, and time spent in administrative tasks, with cost savings per child of £66 (95% CI −93 to −39; p<0·0001), £37 (−53 to −20; p<0·0001), and £13 (−20 to −7; p<0·0001), respectively, in favour of the CBT intervention. The appendix summarises results of cost-utility analyses of the brief guided parent-delivered CBT treatment compared with the solution-focused brief therapy control for the base-case and the ten sensitivity analyses.

**Figure 2:**
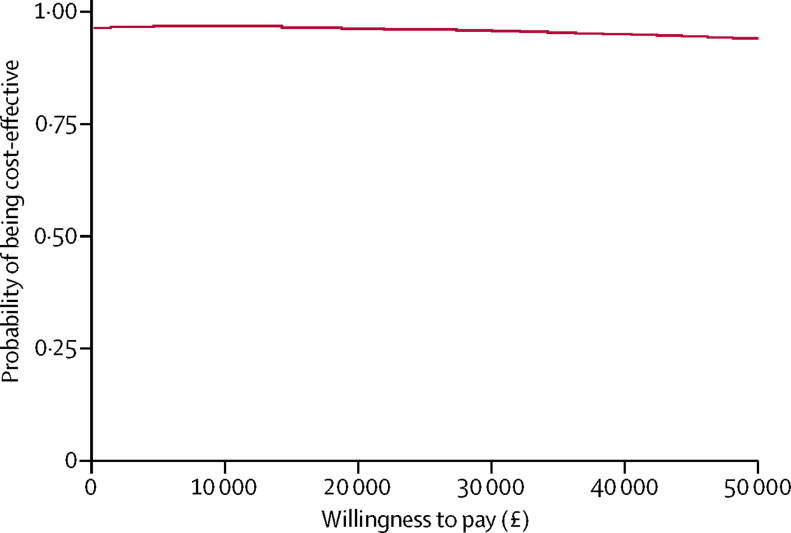
Cost-effectiveness acceptability curve for brief guided parent-delivered cognitive behavioural therapy compared with solution-focused brief therapy for the base-case cost-utility analysis

Taking sampling uncertainty into consideration, the cost-effectiveness acceptability curve for the base-case analysis (figure 2) shows that, in view of the joint distribution of incremental mean costs and effects, the probability that brief guided parent-delivered CBT is cost-effective by comparison with solution-focused brief therapy is around 96%, based on UK National Institute for Health and Care Excellence thresholds for accepted levels of willingness to pay for an extra QALY (usually between £20 000 and £30 000). Sensitivity analyses supported this finding with the probability of brief guided parent-delivered CBT being a cost-effective alternative to standard practice ranging from 74% to 99%.

When brief guided parent-delivered CBT was compared with solution-focused brief therapy in terms of the societal costs per extra child with a CGI-I outcome of much or very much improved (mean difference: effect −0·008, 95% CI −0·160 to 0·144; p=0·92; cost: −£448, −934 to 37; p=0·070), the cost-effectiveness acceptability curve, which accounts for sampling uncertainty, indicated that if the NHS and society were willing to pay £1000 per extra child with a CGI-I outcome of much or very much improved, the probability that brief guided parent-delivered CBT would be cost-effective compared with solution-focused brief therapy would be 96%, and would still be 83% and 57% for a willingness to pay of £5000 and £20 000, respectively. However, the maximum threshold value that society is willing to pay for an extra child with a CGI-I outcome of much or very much improved is unknown.

## Discussion

Treatment outcomes for children with anxiety disorders did not differ significantly according to whether they received brief guided parent-delivered CBT or solution-focused brief therapy. However, when a societal-based cost-effectiveness analysis was done, taking sampling uncertainty into account, brief guided parent-delivered CBT was likely to represent a cost-effective use of resources compared with solution-focused brief therapy. The actual time spent delivering treatment was similar between the two treatment arms, but cost savings were identified across all resource categories in favour of brief guided parent-delivered CBT, particularly in travel costs because of the ability to do phone-based review sessions with parents undergoing brief guided parent-delivered CBT. The differences in administrative costs were unexpected. Further studies are needed to establish whether different methods of delivery of these two treatments would lead to different cost results.

In view of the brevity of both treatments, that the outcomes were similar to those achieved after more intensive (child-focused) CBT approaches is notable. For example, in the large multicentre Child/Adolescent Anxiety Multimodal Study, 60% of children were much or very much improved after 14 h-long sessions of CBT, and 72% at 6 month follow-up,^31^ very similar to the rates in the present study. Additionally, significant improvements were made between the assessments after treatment and at 6 months' follow-up. This finding might suggest that if either treatment is adopted as part of a stepped-care approach to treatment, it might not be necessary to step up all children who do not recover immediately, but to allow a period of monitoring after treatment. Further investigations are needed to help inform decision making about who to step up, when, and, indeed, what they should be stepped up to.

A strength of our study is that it took place within a routine NHS clinical setting. Despite not restricting the study population to children who met formal anxiety disorder diagnostic criteria, 90% did meet such criteria, and baseline scores on parent-report and child-report measures were similar to those reported in studies with populations with anxiety disorders.^8^, ^9^ A development from most previous trials of childhood anxiety treatment is that we compared two active treatment approaches used routinely by the participating therapists.

A further strength of our study is the inclusion of a full cost-effectiveness analysis. Notably, the economic findings were consistent across various sensitivity analyses, and were reinforced when real-world conditions were accounted for, such as larger time gaps between treatment sessions and assessments (ie, sensitivity analyses 1, 2b, 3b, 7b in the appendix). Results based on a restricted health-care provider perspective confirmed the main finding, but showed that costs associated with childhood anxiety disorders would be highly underestimated on this basis (sensitivity analyses 7a and 7b), emphasising the importance of accounting for all costs borne by society in mental health studies. The present study was not powered to detect differences in cost, so further trials are warranted to validate these findings.

Several limitations should be highlighted. The study was powered to detect superiority of one treatment over another and did not aim to allow us to comment on their equivalence. Attrition from the trial meant that we did not achieve the number of participants needed to meet our power calculation. Although representative of many parts of the UK, as a group, participating families were highly educated, affluent, and not ethnically diverse. We focused on children aged 5–12 years, yet only one self-report measure had previously been validated with children under the age of 7 years (the Koala Fear Questionnaire^20^), and we therefore relied heavily on parent report. Our study did not include a waiting-list comparison; however, brief guided parent-delivered CBT has previously been shown to produce better outcomes than a waiting-list control.^8^ This finding, together with the fact that the outcomes were similar to those from other CBT trials, suggests that both interventions were effective. Further investigation will be of interest to examine the mechanisms by which each treatment has its effects. For example, both treatments might have ultimately encouraged children to face fears, test beliefs, or problem solve effectively.

For the economic analysis, although a high percentage of complete data were obtained for treatment resource use (98·2–99·8%), missing data for other resource use varied from 8·8% to 26·5%, and a quarter of participants did not have QALY data at every timepoint. However our sensitivity analyses show that the results are consistent even in the complete-case scenario. Additionally, this study only provides an indication of the short-term cost-effectiveness of brief guided parent-delivered CBT compared with solution-focused brief therapy, and future follow-up assessments are warranted to determine cost-effectiveness in the longer term. Finally, although the costs attributed to school absence for children followed methods used in previous studies,^29^ focusing mainly on government expenditure per pupil, they are likely to underestimate the longer term educational disadvantage that children might experience as a consequence of mental health difficulties.

These limitations notwithstanding, although brief guided parent-delivered CBT was not clinically superior to solution-focused brief therapy, our study provides evidence to support its use as a likely cost-effective, brief psychological approach for treatment of childhood anxiety problems.
