# Supplementary material for: Clinical outcomes and cost-effectiveness of brief guided parent-delivered cognitive behavioural therapy and solution-focused brief therapy for treatment of childhood anxiety disorders: a randomised controlled trial
Source: Lancet Psychiatry. 2017 Jul;4(7):529–39. doi: 10.1016/S2215-0366(17)30149-9 (PMC5483485; doi:10.1016/S2215-0366(17)30149-9)
Supplement: Supplementary appendix [file mmc1.pdf]

THE LANCET
Psychiatry

Supplementary appendix

This appendix formed part of the original submission and has been peer reviewed. We post it as supplied by the authors.

Supplement to: Creswell C, Violato M, Fairbanks H, et al. Clinical outcomes and cost-effectiveness of brief guided parent-delivered cognitive behavioural therapy and solution-focused brief therapy for treatment of childhood anxiety disorders: a randomised controlled trial. *Lancet Psychiatry* 2017; published online May 17. http://dx.doi.org/10.1016/S2215-0366(17)30149-9.

Clinical outcomes and cost-effectiveness of brief guided parent-delivered cognitive behavioural therapy and solution-focused brief therapy for treatment of childhood anxiety disorders: a randomised controlled trial

APPENDIX

Unit costs. All costs in 2013/2014 UK prices

Item	Unit cost (£s)	Source	Notes
Therapist	£38 per hour;<br>£77 per hour of client contact	Pay and Conditions Circular (AforC) 1/2014<br>http://www.nhsemployers.org/case-studies-and-resources/2014/03/pay-and-conditions-circular-aforc-12014<br>(accessed 25/03/15)<br><br>and<br><br>http://www.pssru.ac.uk/project-pages/unit-costs/2014/<br>(accessed 25/03/15)	Annex C: Pay bands and pay points on the second pay spine in England from 1 April 2014. Table 10. Average across Pay bands 5 and 6, and calculated according to the methodology adopted in PSSRU, Unit Costs of Health & Social Care 2014. University of Kent, 2014. Table 12·6 Generic single-disciplinary CAMHS team
Supervisor	£47 per hour;<br>£106 per hour of client contact	Pay and Conditions Circular (AforC) 1/2014<br>http://www.nhsemployers.org/case-studies-and-resources/2014/03/pay-and-conditions-circular-aforc-12014<br>(accessed 25/03/15)<br><br>and<br><br>http://www.pssru.ac.uk/project-pages/unit-costs/2014/<br>(accessed 25/03/15)	Annex C: Pay bands and pay points on the second pay spine in England from 1 April 2014. Table 10. Pay band 7 (spine point 30), and calculated according to the methodology adopted in PSSRU, Unit Costs of Health & Social Care 2014. University of Kent, 2014. Table 9·5 Clinical Psychologist
Mileage allowance	£0·56 per mile	Pay and Conditions Circular (AforC) 3/2014<br>http://www.nhsemployers.org/case-studies-and-resources/2014/07/amended-mileage-rates-from-1-july-2014<br>(accessed 02/03/15)	Table 7. Car (all types of fuel) Annual mileage up to 3,500 miles (standard rate)
Family doctor (GP consultation in	£42	Personal Social Services Research Unit. Unit Costs of Health & Social Care 2014. University of Kent, 2014.http://www.pssru.ac.uk/project-pages/unit-costs/2014/	Table 10·8b. Cost including qualifications, excluding other direct care staff costs.

surgery)		(accessed 13/05/15)	
Social worker	£79	As above.	Table 11·3. Cost per hour of face-to-face contact, including qualifications.
Practice nurse (nurse consultation in surgery)	£13·69	As above.	Table 10·6. Cost including qualifications, and based on duration of contact of 15·5 minutes.
Psychologist	£135	As above.	Table 9·5. Cost per hour of client contact (includes A to E: A. Wages/salary; B. Salary oncosts; C. Qualifications; D. Overheads; E. Capital overheads).
Consultant: psychiatrist	£365·78	Personal Social Services Research Unit. Unit Costs of Health & Social Care 2013. University of Kent, 2013. http://www.pssru.ac.uk/project-pages/unit-costs/2013/ (accessed 13/05/15)	Table 15·7. Cost per-face-to-face contact, including qualifications. Price adjusted for inflation using HCHS 2013/2014 (see also Table16·2. Personal Social Services Research Unit. Unit costs of Health & Social Care 2014. University of Kent, 2014. http://www.pssru.ac.uk./ project-pages/unit-costs/2014/ (accessed 13/05/15)
Community psychiatrist nurse (nurse –mental health)	£74	Personal Social Services Research Unit. Unit Costs of Health & Social Care 2014. University of Kent, 2014.http://www.pssru.ac.uk/project-pages/unit-costs/2014/ (accessed 13/05/15)	Table 10·2. Cost per hour of face-to-face contact (including qualifications)
Education welfare officer	£21·74	Creswell C. et al. 2015. Treatment of childhood anxiety disorder in the context of maternal anxiety disorder: a randomised controlled trial and economic analysis. Health Technol Assess 2015; Vol.19; No.38.	Table 89. Adjusted for inflation using RPI.
Educational psychologist	£39·66	As above.	Table 89. Adjusted for inflation using RPI.
Family liaison officer (school) (approximated with family support worker)	£50	Personal Social Services Research Unit. Unit Costs of Health & Social Care 2014. University of Kent, 2014.http://www.pssru.ac.uk/project-pages/unit-costs/2014/ (accessed 13/05/15)	Table 11·8. Costs per hour of client related work.
Teacher	£37·66	Creswell C. et al. 2015. Treatment of childhood anxiety disorder in the context of maternal anxiety disorder: a randomised controlled trial and economic analysis. Health Technol Assess 2015; Vol.19; No.38.	Table 89. Adjusted for inflation using RPI.
Paediatrician - out-patient attendance: paediatrics	£235	National Schedule of Reference Costs Year: '2013-2014'. https://www.gov.uk/government/publications/nhs-reference-costs-2013-to-2014 (accessed 18/05/15)	National Schedule of Reference Costs Year: '2013-2014' – NHS Trusts. Consultant led outpatient attendances: Non-Admitted Face to Face Attendance, First. Currency Code: WF01B Service code: 420
Audiology – out-patient attendance: paediatric audiological Medicine (A), audiological Medicine (B), audiology (C)	£112·36	As above.	As above. Weighted average of (A), (B) and (C). Currency Code: WF01B- (A): Service code 254 – (B): Service code 310 – (C): Service code: 840
Speech and language (community speech and language therapist)	£36	Personal Social Services Research Unit. Unit Costs of Health & Social Care 2014. University of Kent, 2014.http://www.pssru.ac.uk/project-pages/unit-costs/2014/ (accessed 13/05/15)	Table 9·3. Cost including qualifications.

Ophthalmology – out-patient attendances: Ophthalmology (A), Paediatric Ophthalmology (B), Medical Ophthalmology (C), Orthoptics (D), Optometry (E)	£102·95	National Schedule of Reference Costs Year: '2013-2014'. https://www.gov.uk/government/publications/nhs-reference-costs-2013-to-2014 (accessed 18/05/15)	National Schedule of Reference Costs Year: '2013-2014' – NHS Trusts. Consultant led outpatient attendances: Non-Admitted Face to Face Attendance, First. Currency Code: WF01B Weighted average of (A), (B), (C) and (D). - (A): Service code 130 – (B): Service code 216 – (C): Service code: 460 – (D): Service code 655 – (E): Service code 662
Hospital A&E Department	£108·96	As above.	National Schedule of Reference Costs Year: '2013-2014' – Emergency Medicine: No Leading to Admitted. Weighted average of all Services in the category. Currency codes from VB01Z to VB011Z
Hospital inpatient services - short stay	£1227·95	As above.	National Schedule of Reference Costs Year: '2013-2014' – Weighted average of elective and non-elective inpatients services - short stay
Hospital inpatient services - long stay	£2991·56	As above.	National Schedule of Reference Costs Year: '2013-2014' – Weighted average of elective and non-elective inpatients services - long stay
Day hospital	£698	As above.	National Schedule of Reference Costs Year: '2013-2014' – Day case
Occupational therapist	£36	Personal Social Services Research Unit. Unit Costs of Health & Social Care 2014. University of Kent, 2014.http://www.pssru.ac.uk/project-pages/unit-costs/2014/ (accessed 13/05/15)	Table 9·2. Cost including qualifications.
Paediatric dietician	£37	As above.	Table 13·4. Cost including qualifications.
Paediatric physiotherapist	£36	As above.	Table 9·1. Cost including qualifications.
Paediatric play Specialist	£12·28	Creswell C. et al. 2015. Treatment of childhood anxiety disorder in the context of maternal anxiety disorder: a randomised controlled trial and economic analysis. Health Technol Assess 2015; Vol.19; No.38.	Table 89. Adjusted for inflation using RPI.
Family therapist (family support worker)	£50	Personal Social Services Research Unit. Unit Costs of Health & Social Care 2014. University of Kent, 2014.http://www.pssru.ac.uk/project-pages/unit-costs/2014/ (accessed 13/05/15)	Table 11·8. Costs per hour of client related work.
Community children's nurse	£99	National Schedule of Reference Costs Year: '2013-2014'. https://www.gov.uk/government/publications/nhs-reference-costs-2013-to-2014 (accessed 18/05/15)	National Schedule of Reference Costs Year: '2013-2014' – Community Health Services: Nursing Services for Children – Service code: NURS Currency code: N12
Child & adolescent mental health worker	£69	Personal Social Services Research Unit. Unit Costs of Health & Social Care 2014. University of Kent, 2014.http://www.pssru.ac.uk/project-pages/unit-costs/2014/ (accessed 13/05/15)	Table 12·6. Generic single-disciplinary CAMHS
Primary mental health worker	£69	Personal Social Services Research Unit. Unit Costs of Health & Social Care 2014. University of Kent, 2014.http://www.pssru.ac.uk/project-pages/unit-costs/2014/ (accessed 13/05/15)	Table 12·6. Generic single-disciplinary CAMHS
Housing department	£21·07	Creswell C. et al. 2015. Treatment of childhood anxiety disorder in the context of maternal anxiety disorder: a randomised controlled trial and economic analysis. Health Technol Assess 2015; Vol.19; No.38.	Table 89. Adjusted for inflation using RPI.

Citizens advice bureau	£16·48	As above.	Table 89. Adjusted for inflation using RPI.
Family centre (family support worker	£50	Personal Social Services Research Unit. Unit Costs of Health & Social Care 2014. University of Kent, 2014.http://www.pssru.ac.uk/project-pages/unit-costs/2014/ (accessed 13/05/15)	Table 11·8. Costs per hour of client related work.
Home-start	£98·30	McIntosh E, Barlow J, Davis H, Stewart-Brown (2009). Journal of Public Health. Sep;31(3):423-33.	Table 1, page 427. Price inflated to 2013/14 prices using the HCHS index
Family planning clinic	£71	National Schedule of Reference Costs Year: '2013-2014'. https://www.gov.uk/government/publications/nhs-reference-costs-2013-to-2014 (accessed 18/05/15)	National Schedule of Reference Costs Year: '2013-2014' Outpatient Attendances Data – Total. Service code: FPC
Self-help groups	free	Self Help UK – the guide to patient support and self-help. http://www.self-help.org.uk/search/ (accessed 19/05/15)	There are a variety of self-help groups, support groups and advice line, covering a variety of disease areas, free of charge
Alternative medicine	£50	http://www.nhs.uk/conditions/homeopathy/Pages/Introduction.aspx (accessed 19/05/15)	The price for an initial consultation with a homeopath can vary from around £20 to £80. Average price is here considered.
Advice line	free	Self Help UK – the guide to patient support and self-help. http://www.self-help.org.uk/search/ (accessed 19/05/15)	There are a variety of self-help groups, support groups and advice line, covering a variety of disease areas, free of charge
Other healthcare and social care resource use	£74·79	Authors' calculations.	Average of all other unit costs excluding hospital admissions and day hospital.
NHS prescription costs	BNF1: £5·35<br>BNF2: £3·20<br>BNF3: £16·15<br>BNF4: £9·58<br>BNF5: £4·85<br>BNF6: £12·29<br>BNF7: £12·83<br>BNF8: £53·51<br>BNF9: £11·36<br>BNF10: £6·37<br>BNF11: £6·73<br>BNF12: £6·37<br>BNF13: £7·38<br>BNF14: £ 8·68<br>BNF15: £13·35	Prescription Cost Analysis: England 2013. http://www.hscic.gov.uk/catalogue/PUB17274 (accessed 20/05/2015)	Totals by BNF Chapters
Over-the-counter (OCT) medicines	£2·60	PAGB Fast facts http://www.pagb.co.uk/media/facts.html (accessed 20/05/2015)	OCT medicines: Average product cost
Mother time off-work/leisure (daily rate)	£91·76	Annual survey of hours and earnings, 2013 Revised Results. Office for National Statistics 2014 http://www.ons.gov.uk/ons/rel/ashe/annual-survey-of-hours-and-earnings/2013-revised-results/index.html (accessed 20/05/17)	Table 1·1a - Weekly pay - Gross (£) - For female employee jobs: United Kingdom, 2013: Median gross weekly earning<br>Table 1·9a - Paid hours worked - Total - For female employee jobs: United Kingdom, 2013

Cost of day of school absence	£23·55	School spend per pupil 2012-2013.<br>http://www.education.gov.uk/schools/performance/2013/download_data.html<br>(accessed 20/05/15)	National per pupil medians<br>Income and Expenditure data 2012-2013

Cost Utility Analysis results

Sensitivity analysis (SA)	Cost mean difference	95% CI	p-values	Effect mean difference[a]	95% CI	p-values	Incremental analysis (ICER reported when appropriate)	Probability cost-effective at willingness to pay equal to £20000 per QALY gained[b]	Probability cost-effective at willingness to pay equal to £30000 per QALY gained[c]
Base case	-£448·30	(-£933·66, £37·06)	0.070	0·006	(-0·009, 0·020)	0.42	GPD-CBT treatment dominates	0.964	0·957
SA 1	-£448·30	(-£933·66, £37·06)	0.070	0·038	(-0·004, 0·080)	0.076	GPD-CBT treatment dominates	0.977	0·975
SA 2a	-£448·30	(-£933·66, £37·06)	0.070	0·007	(-0·008, 0·022)	0.34	GPD-CBT treatment dominates	0.984	0·983
SA 2b	-£448·30	(-£933·66, £37·06)	0.070	0·043	(-0·0003, 0·087)	0.051	GPD-CBT treatment dominates	0.993	0·991
SA 3a	-£448·30	(-£933·66, £37·06)	0.070	-0·008	(-0·040, 0·247)	0.65	£56038	0.739	0·642
SA 3b	-£448·30	(-£933·66, £37·06)	0.070	0·022	(-0·030, 0·073)	0.41	GPD-CBT treatment dominates	0.891	0·870
SA 4	-£475·27	(-£960·06, £9·5)	0.055	0·007	(-0·008, 0·021)	0.37	GPD-CBT treatment dominates	0.973	0·967
SA 5	-£468·83	(-£936·37, -£ 1·29)	0.049	0·006	(-0·009, 0·020)	0.42	GPD-CBT treatment dominates	0.971	0·963
SA 6	-£251·49	(-£645·58,£142·60)	0.21	0·002	(-0·014, 0·018)	0.80	GPD-CBT treatment dominates	0.869	0·838
SA 7a	-£133·38	(-£203·69, -£63·07)	<0.0001	0·006	(-0·008, 0·020)	0.42	GPD-CBT treatment dominates	0.952	0·919
SA 7b	-£133·38	(-£203·69, -£63·07)	<0.0001	0·038	(-0·004, 0·080)	0.076	GPD-CBT treatment dominates	0.981	0·975

Footnote: CI Confidence Interval; ICER Incremental Cost Effectiveness Ration; QALY Quality Adjusted Life Years; GPD-CBT Guided Parent Delivered CBT; SFBT Solution Focused Brief Therapy; [a]Adjusted for baseline utility; [b]Probability associated with a threshold of £20000 per QALY gained; derived from the relevant CEAC; [c]Probability associated with a threshold of £30000 per QALY gained; derived from the relevant CEAC; SA 2a -effects measured by CHU9D parent-report ; SA 3a - effects measured by EQ-5D-Y; SA 4 -actual treatment received approach; SA 5 - adjustment for baseline costs; SA 6 - complete-case analysis; SA 7a - healthcare provider perspective; SAs, 1, 2b, 3b, 7b - use of actual time between assessments for estimation of QALYs.

Months between treatment and assessment time points across treatment arms

	GPD-CBT (months) Mean (sd)	SFT (months) Mean (sd)	Mean difference	p-value	95% CI	t-test statistic
Randomisation to end of treatment GPD-CBT (N=59) SFBT (N=66)	3·305 (1·263)	2·955 (1·221)	0·351	0·117	(-0·0895, 0·791)	t(123) =1·577
End of treatment to assessment 1 (post-treatment) GPD-CBT (N=56) SFBT (N=65)	0·661 (0·900)	0·539 (0·812)	0·122	0·434	(-0·186, 0·430)	t(119)=0·786
End of treatment to assessment 2 (6 months post treatment) GPD-CBT (N=56) SFBT (N=62)	5··661 (0·880)	5·677 (1·198)	0·0167	0·932	(-0·403, 0·370)	t(116)=0·086

Footnote: GPD-CBT Guided Parent delivered- Cognitive Behavior Therapy; SFBT Solution Focused Brief Therapy
